# Biotechnological methods as a tool for efficient sugar beet breeding

**DOI:** 10.18699/VJ20.593

**Published:** 2020-02

**Authors:** T.P. Zhuzhzhalova, E.O. Kolesnikova, E.N. Vasilchenko, N.N. Cherkasova

**Affiliations:** A.L. Mazlumov All-Russia Research Institute of Sugar Beet, VNIISS, Ramon raion, Voronezh oblast, Russia; A.L. Mazlumov All-Russia Research Institute of Sugar Beet, VNIISS, Ramon raion, Voronezh oblast, Russia; A.L. Mazlumov All-Russia Research Institute of Sugar Beet, VNIISS, Ramon raion, Voronezh oblast, Russia; A.L. Mazlumov All-Russia Research Institute of Sugar Beet, VNIISS, Ramon raion, Voronezh oblast, Russia

**Keywords:** sugar beet, haploid parthenogenesis, abiotic stresses, selective agents, morphogenesis, micropropagation, stecklings, breeding lines, elite seeds, сахарная свекла, гаплоидный партеногенез, абиотические стрессы, селективные агенты, морфогенез, микроразмножение, штеклинги, селекционные линии, семена элиты

## Abstract

Here we consider aspects of the application of biotechnological methods to rapid creation, propagation, and maintenance of plants with improved or new traits in sugar beet breeding. The results of the works carried out in these fields by the Federal State Budgetary Scientific Institution “The A.L. Mazlumov All-Russia Research Institute of Sugar Beet” are reviewed. A close association between morphological and physiological changes in in vitro cultured organs and tissues, on the one hand, and breeding traits, on the other hand, which allows the development of experimental systems for non-amphimictic plant reconstruction is shown. The influence of in vitro growth conditions on haploid cells of unfertilized sugar beet ovules in the course of obtaining doubled haploid lines with high degree of homozygosity and maintenance of valuable breeding properties is considered. As compared to common inbreeding, this method shortens the time for development of homozygous material from 10–12 to 3–5 years, which is of great importance for speeding-up the breeding process. The results of studies on the culturing of mature sugar beet zygotic embryos based on in vitro selective systems have made it possible to improve the adaptive potential of plants and to provide complex resistance to environmental stress factors. Strict selection under abiotic stress conditions allowed creation of sugar beet isogenic lines with tolerance of drought, salinity, and soil acidity. It is shown that the proposed original design of mass-scale microclonal in vitro reproduction and deposition of elite plants as components of highly productive hybrids can be used to obtain seeds of uniform high-quality breeding material. The technologies developed by biotechnological methods are a topical and innovative direction of inquiry, since the application of these techniques to sugar beet breeding will promote obtaining of competitive hybrids with a set of commercially valuable traits. The combination of biotechnology methods, including tissue culture, and traditional breeding techniques is expected to provide an opportunity to obtain a new starting material to develop domestic varieties and hybrids of new generation with heterosis effect and a wide resistance spectrum persisting across generations.

## Abstract (ru)

Рассмотрены аспекты использования в селекционном процессе сахарной свеклы биотехнологических методов, позволяющих ускоренно создавать, размножать и сохранять растения с улучшенными или новыми признаками. Представлен обзор работ, проведенных по данным направлениям во Всероссийском НИИ сахарной свеклы и сахара им. А.Л. Мазлумова. Показана тесная взаимосвязь морфофизиологических исследований культивируемых in vitro органов и тканей с селекционными признаками, обеспечивающая разработку экспериментальных систем реконструкции растений без полового скрещивания. Рассмотрено воздействие условий культуры in vitro на гаплоидные клетки неоплодотворенных семязачатков сахарной свеклы в процессе получения удвоенных гаплоидных линий с высокой степенью гомозиготности и сохранением ценных селекционных свойств. В отличие от классического инбридинга, время создания гомозиготного растительного материала с помощью данного метода было сокращено с 10–12 до 3–5 лет. Исследования по культивированию зиготических зрелых зародышей сахарной свеклы на основе селективных систем in vitro позволили повысить адаптивные свойства растений и обеспечить комплексную устойчивость к стрессовым факторам внешней среды. Благодаря жесткому отбору в условиях абиотического стресса созданы изогенные линии сахарной свеклы с толерантностью к засухе, засолению и кислотно- сти почвы. Предложена оригинальная схема массового микроклонального размножения и депонирования in vitro элитных растений – компонентов высокопродуктивных гибридов, которая может служить для полу- чения выровненного селекционного материала улучшенного качества. Разработанные технологии являют- ся приоритетным и инновационным направлением исследований, так как внедрение данных разработок в селекционный процесс сахарной свеклы будет способствовать получению конкурентоспособных гибридов с комплексом хозяйственно полезных признаков. Сочетание биотехнологических подходов, в том числе культуры тканей, с методами традиционной селекции даст возможность получать новый исходный материал для создания отечественных сортов и гибридов нового поколения с гетерозисным эффектом и широким спектром устойчивости, сохраняющимися в ряду поколений.

## Introduction

One of the current topical directions in sugar beet breeding is the development of highly productive hybrids on a linear basis. The traditional breeding process involves timeconsuming selection of self-pollinated lines with uniform morphological traits, economically useful properties (yield, sugar content, etc.), and high quality of seeds. However, the breeding methods in use take much time and labor. It is determined by the two-year development cycle of plants, inbreeding depression, and the phenomenon of self- and cross-incompatibility, hampering the maintenance of genetical uniformity in the breeding material. In this connection, biotechnological methods based on in vitro culturing of organs and tissues are promising, as they permit one to develop and stabilize breeding material and to maintain its valuable traits. The application of biotechnologies to the breeding process will allow reduction and then elimination of our lagging behind foreign countries in different indices of agricultural industry.

Raise of sugar beet doubled haploids in tissue culture will produce homozygous lines to obtain hybrids with a set of commercially useful traits. This is an important stage of sugar beet breeding, which should be finished and included in the technological process. Use of in vitro selection systems promoting the breeding of plants with high resistance to abiotic stresses, which is based on the ability of vegetative organisms to develop mechanisms of protection from adverse environment factors at the cell level (Dukhovsky et al., 2003; Lamaoui et al., 2018) is also promising in sugar beet industry.

In practical sugar beet breeding, the refinement of biotechnological methods for mass reproduction and long maintenance of valuable forms is one of the main directions in works with isolated tissue cultures. An important basis of these methods is the unique property of totipotency in plant cells characterizing the whole potential of traits of an individual, which, when cultivated, is implemented through different ways of morphogenesis: embryoidogenesis, organogenesis, and histogenesis (Batygina, 2000). In this connection, the morphogenetic competence of cultivated tissue and organs of sugar beet plants can promote a certain way of development, which demands special attention of researchers when microcloning the developed material.

To carry out research in the above-mentioned directions, a number of international scientific institutions have been involved. In particular, to improve sugar beet germplasm, methods for development of doubled haploids, estimation of resistance to stresses, various molecular methods, and so on are used in the United States. The Agricultural Research Service of the United States Department of Agriculture (USDA ARS) supervises all studies (Kozlovsky et al., 2016). The international interest to haploid plants in the European Union is confirmed by development of EU GOST Action 851 “Gametic cells and molecular breeding for crop improvement”. Homozygous lines are used in all breeding and seed-growing companies in Europe such as Strube, KWS, Syngenta, and Fiorimond Despres, engaged in the development of sugar beet hybrids and seed material. The development of sugar beet haploid and doubled haploid forms (x/xx) is an integral part of investigations at the Planta research center. There, basic investigations in different fields of biotechnology including working out of methods of sugar beet breeding for resistance to stresses are concentrated. Using biotechnologies, sugar beet breeding materials were obtained in Germany, Turkey (Gürel et al., 2003), Poland (Tomaszewska-Sowa, 2012), Bulgaria (Kikindonov et al., 2016), and so on. Technological indices of the breeding material produced with the help of sugar beet doubled haploids in Byelorussia, which demonstrate success in breeding at (or above) the level of usual hybrids, are of interest. Tests of the doubled haploids showed that they exceeded the diploid standard in beet root yield (43–45 t/hectares) and sugar content (20.9 and 21.1 %) (Kilchevskiy, Hotyleva, 2012). A long-term study of hybrids involving DH lines produced in Bulgaria showed high performance as compared to traditional hybrids. As for beet root yield, dihaploid hybrids exceeded the control by 11.4–13.7 %, and the sugar yield was about the standard level (Kikindonov et al., 2016). These results are of practical importance, as they promote the introduction of technologies based on tissue culture methods for rapid raise of breeding material with valuable traits to be included into breeding programs.

In connection with the above, investigations in the development and introduction of biotechnologies for targeted production of genetically improved starting material for domestic sugar beet breeding in order to produce newgeneration promising hybrids are topical. Success of these investigations can be ensured if taking into account specific features of morphogenetic potentialities of the development of certain plant organs tending to intense regeneration and reproduction under in vitro conditions.

## Haploid parthenogenesis

In the course of long-term investigations at the Federal State Budgetary Scientific Institution “The A.L. Mazlumov All-Russia Research Institute of Sugar Beet”, methods of in vitro haploid parthenogenesis induction were developed, and optimum conditions of sugar beet explants’ culture were determined (Vasilchenko et al., 2018). The choice of initial plants as donors of explants with regard to phenotype features, including the morphological habitus of seed-bearing plants, high degree of seed monogermity, and seed setting, is an important issue. Ovules located in buds in the central ear of the pleiochasium cluster were primary explants for in vitro culturing. Cytoembryological studies (Barykina et al., 2004) indicated that in vitro formation of haploid embryoids occurred at all development stages of embryo sac nuclei and cells. It was promoted by pronounced polarity and high ability of haploid cells to develop. Ovules containing eight haploid nuclei or seven cells (but also octonuclear) in embryo sacs displayed the greatest activity in the formation of haploid embryoids. According to the present notion, the switch of the development program from the gametophyte to sporophyte type can occur in this time span (Batygina, Vasilyeva, 2002). Exposure of material to low positive temperatures during 2–4 days stimulates this process.

The method using alternation of В5 nutrient media differing in consistence proved to be the bottom line in ovule culturing. High gibberellin concentrations in the liquid nutrient medium caused direct regeneration of haploid embryoids with embryo root and cotyledon leaves formation. Transplantation to a solid nutrient medium was followed by the formation of continuance shoots. Presence of 6-BAP in a liquid medium induced the formation of callus tissue, whose transplantation to a solid nutrient medium with addition of kinetin (Kn) and 2.4-D induced the development of regenerants by hemmorhizogenesis. In this technique, the cultivated explants retained their viability for up to 4–6 months and the proliferation and differentiation processes were activated in female gametophyte nuclei. The specialization of the formed cells of plant tissues increased the output of haploid regenerants by up to 18.9 % through direct embryoidogenesis and by up to 11.0 % by callusogenesis. Such method accelerated cell differentiation by 25–30 % and increased the number of the formed regenerants by up to 13–19 % (Vasilchenko, 2016).

At the next step, morphologically normal haploid regenerants were selected and transformed to diploids in colchicine-containing nutrient medium. All stages of the process were accompanied by selection for cytological, biochemical, and molecular traits (Zhuzhzhalova et al., 2016). Raise of haploid plants has long been known. Ploidy was most often determined by directly counting chromosomes in a nucleus (Nitzsche, Wenzel, 1980; Pausheva, 1988). Analysis of nDNA by flow cytometry based on fluorescent staining of plant nuclei (Tomaszewska-Sowa, 2010) provided an opportunity to select regenerants with the haploid chromosome set (n = 9) rapidly and efficiently, and, after treatment with colchicine, to select doubled haploids (2n = 18). It should be noted that cell division aberrations were often observed after mutagenesis; this resulted in varying chromosome quantities in regenerants and occurrence of mixoploid and aneuploid forms, which were rejected (Zhuzhzhalova et al., 2016).

Biochemical evaluation revealed different enzyme activity patterns in experimental specimens. In comparison with initial diploid forms, haploid plants were characterized by the protein quantity elevated by 60 % and greater activities of peroxidase, glucose-6-phosphate-dehydrogenase, and isocytrate-dehydrogenase. After chromosome doubling in regenerants, these values returned to the control or slightly higher levels (Zemlyanuhina et al., 2016). It is conjecturable that the change in enzyme activities in sugar beet haploid regenerants was due to methylation of DNA genome sites connected with protein functioning. The revealed differences reflected more profound changes caused by cell differentiation during tissue culturing (Vanyushin, 2010). This issue demands a special comprehensive study.

After diploidization of regenerants, the detection of hereditary changes not expressed phenotypically was of great importance. РСR and RFLP analyses using Hind III restrictase (Beckman, Sollen, 1983) made it possible to identify the cytoplasm type from the number of fragments after chromosome doubling. In microclones with normal cytoplasm, one fragment (800 bp) was amplified. In forms with the sterile type of cytoplasm, two digestion products, 320 and 480 bp in length, were revealed. Doubled haploids in which this fragment was not digested by Hind III included completely fertile forms with normal cytoplasm (N) and nuclear genes in the recessive form (rf). In other specimens, polymorphism of fragments was observed, which supposed the presence of sterile cytoplasm (S) and different combinations of recessive and dominant alleles of the nuclear genes Rf1/rf1 and Rf2/rf2 in the corresponding haploid forms. The identification of cytoplasm type in regenerants at early steps of culturing facilitated the development of lines with CMS, being of considerable interest for sugar beet breeding (Vasilchenko, 2016; Vasilchenko et al., 2018).

The last step of in vitro culturing included the cloning of regenerants with doubled ploidy and their subsequent rooting on a nutrient medium with modified hormonal composition. The selected microclones with root systems and well-developed leaf surface were planted to soil under greenhouse conditions. Keeping air humidity high allowed up to 72 % survival of microclones. Further, plants were grown for 3 months till obtaining stecklings of 70–100 g in weight, which were then vernalized for 45 days. At the beginning of seedstalk formation, the greenhouse air temperature was raised to 14–20 °C, and constant illumination was kept for a month. In this time, intense growth and development of seed-bearing plants were noted. Then, budding and flowering were observed. The heights of plants were 1.5 meters or more, and the thicknesses of the main flower-bearing stems were about 3.0 cm. Flowering was synchronous and intense. The fertility of pollen grains varied from 87 to 92 %. In 8–10 weeks, seeds with a high level of monogermity were formed.

Our study revealed the basic conditions for inducing haploid parthenogenesis and allowed a three-year cycle for producing homozygous DH lines to be elaborated. The obtained seeds of four DH lines became a new starting material, and now they are used in breeding work. The development of sugar beet homozygous lines will promote rapid raise of hybrids with a set of economically valuable traits (Kolesnikova et al., 2018b).

## In vitro selective systems

As a result of our experiments, a method of in vitro selection of sugar beet forms was originated in order to develop unique breeding material with resistance to adverse environmental conditions (Cherkasova, Zhuzhzhalova, 2011). The method is based on double passage of regenerants under severe selective conditions, after which the regenerants surviving high chloride concentrations constituted 66.0–81.7 %. Further passage of salt-tolerant forms on a nutrient medium supplemented with a nonionic osmotic component (0.45 M) promoted an improvement of adaptation mechanisms of plant protection under osmotic stress conditions. In this case, the survival rate of genotypes doubled, and the level of general regenerant adaption to abiotic stress (drought + salinization) reached 59.0 %. The passage of the obtained regenerants at elevated acidity (рН 3.5) was accompanied by intense development of continuance shoots, which pointed to a high resistance of microclones, 69.1–87.5 %. The level of general adaption of regenerants to a more complex stress (drought + acidity) increased tenfold (Cherkasova et al., 2018).

When developing the method, of great importance were the experiments that demonstrated that the growth of unspecialized plant tissues (petioles, shoot buds, immature embryos, etc.) on selective nutrient media with addition of sea salt (2 %) and sorbitol (0.45 M at рН 4.0) led to almost complete degradation of plant material. During primary selection, the emergence of regenerants with resistance was observed only in 2–4 % of explants, for which mature embryos were used. This period was accompanied by slow formation of continuance shoots in the survived explants. It was connected with retardation of cell extension and division processes, which resulted in inhibition of regenerant development (Shabala, Munns, 2017). Use of secondary selection, which, in addition, increased the degree of resistance in the regenerants under severe selection conditions, proved to be efficient.

Further mass-scale systemic selection of regenerants according to the root length index (Kosareva, 2012), which varied from 1.0 to 1.2 in the experiments, increased the percentage of survived tolerant plants to 87.5–89.2 %. The regenerants selected according to root length under conditions that simulated high soil acidity (рН 3.5) and drought were remarkable for well-developed root system along with the uniformity of leaf apparatus. Root formation under conditions of osmotic stress was less changeable than leaf development. After the exposure to osmotic components, the rate of root elongation restored quickly. The intense development of the root system under conditions of osmotic stress was determined by the acceleration of lateral root growth, providing better access to water and nutrients (Rahnama et al., 2011).

The revealed features of the morphological development of sugar beet regenerants were associated with changes in a variety of biochemical processes that enhanced the work of oxidative stress enzymes, increased protein synthesis rates (Zemlyanuhina et al., 2017), and caused accumulation of free proline (Bates еt al., 1973).

Under conditions of water deficiency, proline increased osmotic pressure of cell juice, which was indicative of its osmoregulation function (Soshnikova et al., 2013). Under abiotic stress conditions, the accumulation of proline (from 36.9 to 79.3 microgram/g) in salt-tolerant sugar beet regenerants was associated with the genotype. The resistance coefficient K, determined as the ratio between the quantity of proline at stress and its initial content, was 5.8–10 times more in salt-resisting forms than in the control. Further passage of salt-tolerant forms with the presence of sorbitol at concentrations 0.45–0.40 М increased the resistance coefficient by a factor of 1.5–1.9. During the systemic selection (drought + salinization), the overall osmotic resistance of sugar beet genotypes increased by a factor of 7–12. These data allowed proline quantity to be used as an index of sugar beet regenerant adaptation to stress and as an indicator of salt-resistant components (Cherkasova, Zhuzhzhalova, 2014).

At the final stage, the selected forms with well-developed root systems were grown under greenhouse conditions to obtain stecklings and then seed-bearing plants.

These methods make it possible to carry out selection of sugar beet forms with resistance to edaphic environmental factors to produce isogenic lines within 3–4 years instead of the previously required 8–10. It is of great importance for breeding process when developing new sugar beet hybrids tolerant of abiotic stresses.

## Micropropagation

It has been shown that in the course of the development of stress-resistant isogenic lines cultured sugar beet petioles, or leaves of young shoots, or embryos produce a new organism through embryoid formation (Zhuzhzhalova et al., 2006). The key difference between the emerging embryolike bodies and haploid embryoids is the formation of the former from diploid somatic cells of plants without participation of sexual gametes. The morphogenesis was based on the formation of initial cells, having dense cytoplasm and a large nucleus, in epidermis. In the next division, a two-cell proembryo formed to produce then a spherical somatic embryoid. At the next stage, the formation of a heart-shaped embryoid and a shoot with development of a stem with cotyledon leaves was observed. Apparently, the stressful situation created by in vitro conditions caused morphogenesis implementation via somatic embryoid formation. The resulting gametophytic and somatic embryoids, irrespective of their origin, were new organisms in their nascence and were primitives of plant reproduction by analogy with meristematic cells (Batygina, Vasilyeva, 2002; Batygina et al., 2010). In this connection, the stage of embryoid induction is the key step of in vitro micropropagation of the produced sugar beet plant material.

It is known that sugar beet is reproduced under natural conditions solely by seeds and that it has only one backup mode to survive: vegetative propagation by traumatic partition. This method was widely used in the breeding practice of the 20th century for vegetative propagation of beet roots serving as ancestors of valuable starting material with maintenance of their most important traits: high sugar content, productivity, beet root shape, and so on. Now, it has been proven that types, methods, and forms of seed reproduction can be responsible for the reproductive ability of any plant species in combination with vegetative reproduction (Batygina et al., 2010). Only those species are the most productive in which generative and vegetative reproduction are combined. Therefore, the use of plant propagation in sugar beet by microcloning may improve its reproductive ability, seed production, and other valuable traits and properties.

At present, in vitro micropropagation on the basis of meristem culture is the main method both for the rapid propagation of valuable breeding materials, and for their long-term maintenance (Zhuzhzhalova et al., 2018). By using microcloning in practical breeding, a variety of initial lines with traits of CMS fixing ability, smooth surface of a beet root, great quantity of seeds on seed-bearing plants and so on can be maintained in culture, propagated, and employed in the breeding process (Znamenskaya, 2010).

Studies on using micropropagation and in vitro deposition to improve the morphogenetic potential of hybrid components produced according to the three-line breeding design were of considerable practical interest. The involvement of tissue culturing in the breeding process provided an increase in the reproductive ability of the developed hybrid components and brought about high-grade sugar beet seeds (Kolesnikova et al., 2018a).

At the first step, elite genotypes were selected from three components of a highly productive hybrid: lines with cytoplasmic male sterility, a pollinator of O-type with the CMS fixing ability, and a fertile pollinator able to stimulate the heterosis effect in F1. When selecting the genotypes, use was made of instant diagnostics with regard to morphologic and cytoembryologic traits reflecting developmental stages of seed-bearing plants, mono-/multigermity, pollen grain fertility, synchrony of flowering, ploidy, etc. For in vitro culture introduction of the selected genotypes, apexes of 10–20 floral shoots from the same seed-bearing plant were used. The main inducers of continuance shoot development from apical meristem were hormonal components of the Gamborg’s and Murashige–Skoog nutrient medium (Butenko, 1964). The presence of growth hormones – 6-benzylaminopurine, kinetin, and gibberellin – in the growth medium caused the formation of up to eight adventive shoots per one explant, and the height of the regenerants was about 60–73 mm. At the end of stabilization, the formed regenerants had deep green leaves, the optimal ratio between the petiole and leaf blade, good stooling, and developed apical point. Such microclones successfully formed many axillary branches and then sufficient numbers of elite CMS, O-type, and multigerm pollinator regenerants (Kolesnikova, Zhuzhzhalova, 2018).

The required number of morphologically developed regenerants of hybrid line components were simultaneously in vitro cultivated in a collection of elite clones by deposition. It allowed long-term maintenance of hybrid line component purity at the level of the first generation. The regenerants were cultured without transplantations on hormone-free nutrient medium with high agar content at weak illumination (500–600 lx), and the 16L:8D light schedule.

The second stage included mass-scale micropropagation, rooting of the regenerants, and raise of hybrid component stecklings. For mass propagation, the deposited elite regenerants were transplanted to hormonal В5 nutrient medium, which activated meristems and induced the proliferation of lateral shoots. Repeated replanting of shoots provided the sufficient number of microclones. The greatest number of regenerants was obtained from monogerm fertile lines (48 shoots per explant on the average), and MS-lines and pollinators gave the least ones (16 and 13 shoots, respectively). It should be noted that forms with CMS appeared to be very sensitive to changes in culturing conditions and died more often than fertile genotypes. They were more difficult to stabilize under in vitro conditions and to reproduce later on (Kolesnikova et al., 2018a).

The regenerant rooting was associated with the induction of adventitious roots, achieved by modification of the nutrient medium hormonal composition. Root formation lasted 3–4 weeks. The resulting sugar beet microclones with strong root systems and well-developed above-ground parts were transferred to nonsterile conditions of soil substrate, where adaptation of the microclones was conducted. The microclone survival, growth, and development during the adaptation period were determined by the humidity conditions for the microclones. The keeping of high air humidity allowed 80–100 % survival of microclones. The plants formed in a greenhouse were characterized by uniformity of morphological traits within a line. The developed plants of hybrid line components were grown under greenhouse conditions till the formation of stecklings. The final important process of this step was the harvesting of stecklings and their vernalization by storing in refrigerators.

At the third stage, breeders used the seed-bearing plants obtained from stecklings for hybridization under field conditions, in separate field plots according to the schemes used to obtain seeds of a simple hybrid and a multigerm pollinator of improved quality (Zhuzhzhalova et al., 2017).

The elaboration of biotechnological methods for massscale in vitro reproduction and deposition of sugar beet breeding materials is innovative. It is of top priority and great importance, as it makes it possible to obtain sugar beet lines with high genetic uniformity and better seed material. The introduction of this method, which speeds up breeding twofold, would add much to the development of highly productive hybrids for commercial use and in seed industry, providing maintenance of high indices of economically useful properties of the developed breeding material.

## Conclusion

In this review, we describe the main biotechnology methods presently used to obtain and propagate new material for sugar beet breeding. Haploid parthenogenesis is a novel method promising for breeding programs. Eliminating the necessity of multiple self-pollinations in plants, the method halves the time required for the raise of homozygous material with valuable traits. Lines of doubled haploids have been produced and monogerm seeds of sugar beet hybrid components have been obtained on its basis.

Studies that have enabled the development of in vitro breeding principles making sugar beet regenerants more tolerant of great salinization, drought, and soil acidity are of vital importance. The obtained results hold remarkable promise for breeding, as the approach under consideration allows the development of isogenic lines with high osmotic resistance to adverse environmental factors within three years.

Mass-scale micropropagation and in vitro deposition of elite hybrid components using the three-line breeding scheme is helpful in sugar beet breeding. This method not only increases the productive ability of seed-bearing plant, but also produces high-grade sugar beet seeds.

Introduction of these technologies into sugar beet breeding and seed industry is a focal and innovative direction of studies enabling increase of the homozygosity level in lines and improvement of seed material quality with maintenance of high performance in the developed hybrids.

## Conflict of interest

The authors declare no conflict of interest.
